# RNA binding protein: coordinated expression between the nuclear and mitochondrial genomes in tumors

**DOI:** 10.1186/s12967-023-04373-3

**Published:** 2023-07-28

**Authors:** Jiaoyan Ma, Liankun Sun, Weinan Gao, Yang Li, Delu Dong

**Affiliations:** 1 Department of Pathophysiology, College of Basic Medical Sciences, Jilin University, Changchun, 130021 China; 2 Department of Physiology, College of Basic Medical Sciences, Jilin University, Changchun, 130021 China

**Keywords:** OXPHOS, Cytoplasmic translation, Mitochondrial translation, Retrograde signals, LLPS

## Abstract

Mitochondria are the only organelles regulated by two genomes. The coordinated translation of nuclear DNA (nDNA) and mitochondrial DNA (mtDNA), which together co-encode the subunits of the oxidative phosphorylation (OXPHOS) complex, is critical for determining the metabolic plasticity of tumor cells. RNA-binding protein (RBP) is a post-transcriptional regulatory factor that plays a pivotal role in determining the fate of mRNA. RBP rapidly and effectively reshapes the mitochondrial proteome in response to intracellular and extracellular stressors, mediating the cytoplasmic and mitochondrial translation balance to adjust mitochondrial respiratory capacity and provide energy for tumor cells to adapt to different environmental pressures and growth needs. This review highlights the ability of RBPs to use liquid–liquid phase separation (LLPS) as a platform for translation regulation, integrating nuclear–mitochondrial positive and retrograde signals to coordinate cross-department translation, reshape mitochondrial energy metabolism, and promote the development and survival of tumor cells.

**Supplementary Information:**

The online version contains supplementary material available at 10.1186/s12967-023-04373-3.

## Introduction

The Warburg effect proposes that cancer cells primarily use glycolysis for adenosine triphosphate (ATP) production. However, a meta-analysis of 31 cancer cell lines revealed that oxidative phosphorylation (OXPHOS) contributes an average of 83% to ATP production in cancer cells, indicating that OXPHOS plays a crucial role in cancer progression [1, 2]. Metabolic reprogramming is a hallmark of cancer, giving rise to a mixed glycolysis/OXPHOS phenotype that improves cellular adaptability and provides the necessary conditions for rapid growth and proliferation [3–5]. As vital organelles, mitochondria are necessary for cancer cell proliferation and tumorigenesis, as recently demonstrated through metabolomics and metabolic flux analyses [6]. Additionally, the mitochondrial OXPHOS dependence of tumors forms the basis for the development of chemoresistance and radioresistance [1, 7]. Thus, cancer cells regulate mitochondrial translation to adaptively modulate mitochondrial function and switch between oxidative phosphorylation and glycolysis, allowing survival under fluctuating environmental and stress conditions and promoting tumor progression under conditions such as nutrient deprivation, hypoxia, and drug stimulation [8–10].

Translation is considered the most energy-consuming cellular biological process. Therefore, the precise regulation of translation enables the efficient utilization of energy and the maintenance of cell adaptation to the environment. Eukaryotic cells have two independent translation systems, cytoplasmic and mitochondrial, and the coordination between these two translation systems is a manifestation of the cross-compartmental regulation between the cell nucleus and mitochondria. Studies suggest that the balance of OXPHOS subunit translation encoded by nDNA and mtDNA can ensure the precise assembly and proper function of OXPHOS complexes, maintaining mitochondrial protein homeostasis and allowing tumor cells to adapt to energy demands [11]. When cells face internal or external stress, cytoplasmic translation can be enhanced or reduced, and nuclear (forward) or mitochondrial translation can also act as a signaling sensor to transmit signals back to the nucleus, precisely adjusting the regulatory role of the nucleus on the mitochondria, which in turn regulates the stability of mitochondrial proteins, allowing the oxidative phosphorylation system to function as a backup energy production system in tumor cells, producing and regulating ATP in a finely tuned manner [12, 13].

Increasing evidence suggests that tumor cells promote the aberrant expression of oncogenic proteins by hijacking the translation machinery, which activates oncogenic signaling pathways and contributes to tumorigenesis, progression, and drug resistance. Therefore, targeting translation has emerged as a promising approach for cancer therapy [14]. The PI3K/mTOR signaling pathway is one of the most frequently dysregulated pathways in cancer, with mTOR effectively integrating metabolic information, regulating mRNA translation, and coordinating energy expenditure and mitochondrial energy production during translation [15–17]. mTOR-driven translation regulation depends primarily on the selective modulation of specific target mRNAs by eukaryotic translation initiation factor 4E (eIF4E) binding proteins and RNA-binding proteins, such as la-related protein 1 (LARP1) [18]. RNA-binding proteins are critical regulators of RNA metabolism, influencing protein expression by modulating processes such as RNA splicing, polyadenylation, mRNA stability, localization, and translation. Aberrant RBP expression in tumors is closely linked to tumor cell proliferation, invasion, growth, and drug resistance. RBPs may function as upstream coordinators of translation regulation, dynamically balancing cytoplasmic and mitochondrial translation and serving as sensors that receive signals and ultimately provide feedback on translation regulation, altering downstream target mitochondrial-related proteins to reshape translation and alter tumor metabolic flexibility, ultimately affecting cancer development.

### Mitochondrial gene expression in cancer cells

The uncontrolled proliferation and growth of cancer cells rely on energy production and protein synthesis. During the process of cancer initiation and progression, tumor signaling pathways and external microenvironmental stresses can adaptively regulate protein level changes and reshape translation, driving tumor formation and malignant transformation [19]. Protein synthesis is the most energy-consuming cellular process, and tumor cells continuously adapt to cellular conditions and microenvironments by regulating energy production according to energy demands [17]. Most energy is generated through efficient mitochondrial OXPHOS [20]. Mitochondria are symbiotic organelles derived from α-proteobacterial and ancient archaeal host cells and have selectively retained their bacterial ancestral genome and gene expression machinery. Human mitochondria contain over 1100 proteins regulated by both mitochondrial and nuclear genomes, thus achieving a more complex balance of translation control [21]. Twenty-five percent of mitochondrial proteins maintain and express 13 key proteins, further demonstrating the importance of the coordinated expression of the two genomes in the different cellular compartments [22]. Mitochondrial DNA encodes 37 gene products, including the two ribosomal RNAs (rRNAs) and 22 transfer RNAs (tRNAs) necessary for mitochondrial translation and 13 OXPHOS subunits that drive oxidative phosphorylation and affect energy conversion [23]. Mitochondrially encoded NADH dehydrogenase subunit 1 (MT-ND1)-MT-ND6 and MT-ND4L are subunits of complexI, mitochondrially encoded cytochrome b (MT-CYB) is a subunit of complex III, mitochondrially encoded Cytochrome c oxidase 1 (MT-COX1)–MT-COX3 are subunits of complex IV, and mitochondrially encoded ATP synthase 6(MT-ATP6) and MT-ATP8 are subunits of ATP synthase (complex V) [24]. All complex subunits, except for mitochondria-encoded proteins, are encoded by nuclear DNA and transported to the mitochondria through the mitochondrial transport system to perform their functions [25]. The synthesis of these mitochondrial-encoded proteins requires specialized mitochondrial proteins to complete the transcription and translation process, indicating that the maintenance of mitochondrial translation depends on cytoplasmic translation supported by nuclear regulation. These two translation systems coordinate with each other to achieve the chemical assembly of OXPHOS complexes. The similarities and differences between cytoplasmic protein translation and mitochondrial protein translation, as well as their translation processes,  are shown in Additional file 1. Previous studies have shown that the growth rate of tumor cells decreases when mitochondrial DNA is depleted [26], and restoring the respiratory function of mitochondrial genomes can recover the tumorigenic potential of cancer cells. Inhibiting electron transport chain (ETC) genes can render cancer cells sensitive to glucose depletion, thus slowing tumor progression [27–29]. In recent years, antibiotics have been used to interfere with mitochondrial translation to target cancer cell bioenergetic metabolism, resulting in mitochondrial dysfunction. All of these findings demonstrate the crucial importance of the correct translation of OXPHOS complexes in tumor cells.

The quantity of RNA and the translation rate determine the protein level The relationship between OXPHOS abundance and RNA and translation has been investigated by analyzing data collected from both mitochondrial and cytoplasmic ribosomes. The results indicated that translation regulation of mitochondrial OXPHOS subunits might be the key to their function because the balance of OXPHOS subunits in each compartment was regulated by translation, but RNA levels were weakly correlated [30]. In the past decade, research has revealed a critical role of mitochondrial translation and mitochondrial ribosomal proteins in the prevention of apoptosis, the survival of cancer cells, and the potential of these proteins to serve as biomarkers [31]. Mitochondrial gene expression can be modulated by altering mRNA stability and ribosome activity, and the plasticity of mitochondrial protein translation can be regulated selectively in response to various environmental factors [32]. The quick and timely management of the OXPHOS system from the translation level of mitochondrial protein is a backup system for the regulation of energy generation. This system is important for tumor growth, metabolism, and medication resistance.

The control of mitochondrial translation varies between tumor and non-tumor cells [33]. Antibiotics that impede mitochondrial translation to target mitochondrial protein production can destroy several types of cancer stem cells [34]. The bacterial antibiotic quinupristin/dalfopristin (Q/D) has been shown to bind to mitochondrial ribosomes, prevent mitochondrial protein synthesis, and disrupt OXPHOS, all of which prevent the development of glioma stem cells [35]. Doxycycline therapy reduces A549 cell growth. The protein levels of 13 polypeptides encoded by mitochondrial DNA have been identified using 35S radioactive labeling, and the findings suggest that the anticancer impact of doxycycline in *vivo* is due to its suppression of mitochondrial protein synthesis. This inhibition of mitochondrial protein synthesis decreased mitochondrial energy generation [36]. The above studies show that growth and survival of tumor cells can be affected by the inhibition of mitochondrial translation.

Blocking mitochondrial translation can enhance the sensitivity of tumor cells to chemotherapy. Wang et al. demonstrated that doxycycline significantly decreased the activity of mitochondrial complexes I, III, IV, and V in 786-O cells, but had no effect on complex II, which lacks mitochondrial coding subunits. It is hypothesized that doxycycline affects renal cancer cells by selectively inhibiting mitochondrial DNA translation, destroying various mitochondrial complexes, impairing mitochondrial respiration, and making cancer cells more susceptible to chemotherapy [37]. Hu et al. demonstrated that tigecycline specifically inhibits mitochondrial ribosome translation in the ovarian cancer cell lines SW626 and SK-OV-3. This effect results in mitochondrial dysfunction, oxidative stress and injury, activation of AMP-activated protein kinase (AMPK), and inhibition of mTOR signaling in ovarian cancer cells. The authors used electron transport chain inhibitors, DNA depletion, and pyruvate and uridine rescue. It was demonstrated in three different ways that the key factor lowering cell survival was tigecycline’s inhibition of cell respiration. Thus, it is possible to treat ovarian cancer resistance by using tigecycline to inhibit mitochondrial translation [38]. Tan et al. discovered that tigecycline specifically inhibits mitochondrial translation-induced mitochondrial dysfunction and oxidative damage, selectively targeting hepatocellular carcinoma cells and significantly enhancing the inhibitory effect of the chemotherapeutic drug cisplatin on hepatocellular carcinoma in vitro and in *vivo* [39].

Abnormal expression of mitochondrial translational factors and mitochondrial ribosomal proteins (MRPs) is increasingly associated with cancer. Various metabolic problems that are closely linked to aging and cancer can result from mitochondrial ribosome translation disorders. Zhu et al. discovered that mitochondrial translation elongation factor 4 (mtEF4) overexpression promotes the growth of cancer, but mtEF4 deletion causes abnormalities in the respiratory chain complex and apoptosis. This finding suggests that controlling the translation of mitochondrial-encoded proteins can influence tumorigenesis [40]. The upregulation of mitochondrial translation initiation factor mitochondrial translation initiation factor 2 gene (MTIF2) is also associated with poor prognosis in inorganic arsenic-induced malignant tumors. This is because MTIF2 helps regulate the expression of genes involved in mitochondrial translation, and its upregulation can stimulate the growth and proliferation of cancer cells [41]. Mitochondrial ribosomal subunit dysregulation has been observed in numerous malignancies. By modulating mitochondrial translation, this dysregulation changes tumor metabolism and increases tumor heterogeneity, invasion, treatment resistance, and metastasis [31, 42]. The high expression of MRPL15 and MRPL38 indicates poor prognosis in non-small cell lung cancer and ovarian cancer [43, 44]. The expression of MRPL13 in breast cancer is significantly higher than that in normal tissues, and it can promote tumor cell proliferation and migration through the PI3K/Akt/mTOR signaling pathway [45]. The overexpression of MRPS6 and MRPS23 can impact the breast cancer tumorigenic process [46]. By modulating OXPHOS, methylation of MRPS23 increases breast cancer metastasis [47]. MRPS22, MRPS34, and other diseases destabilize small ribosomal subunits, leading in diminished mitochondrial translation and OXPHOS deficiency [48, 49]. These studies have shown that mitochondrial translation is required for functional mitochondria. And the coordinated expression of nucleus and mitochondrial genome is necessary to maintain mitochondrial function, which suggests that it can play a key role in cancer plasticity, metastasis and drug-resistance, and is a potential target for drug development (Additional file 1).

### RNA-binding proteins in cancer cells

**Fig. 1:**
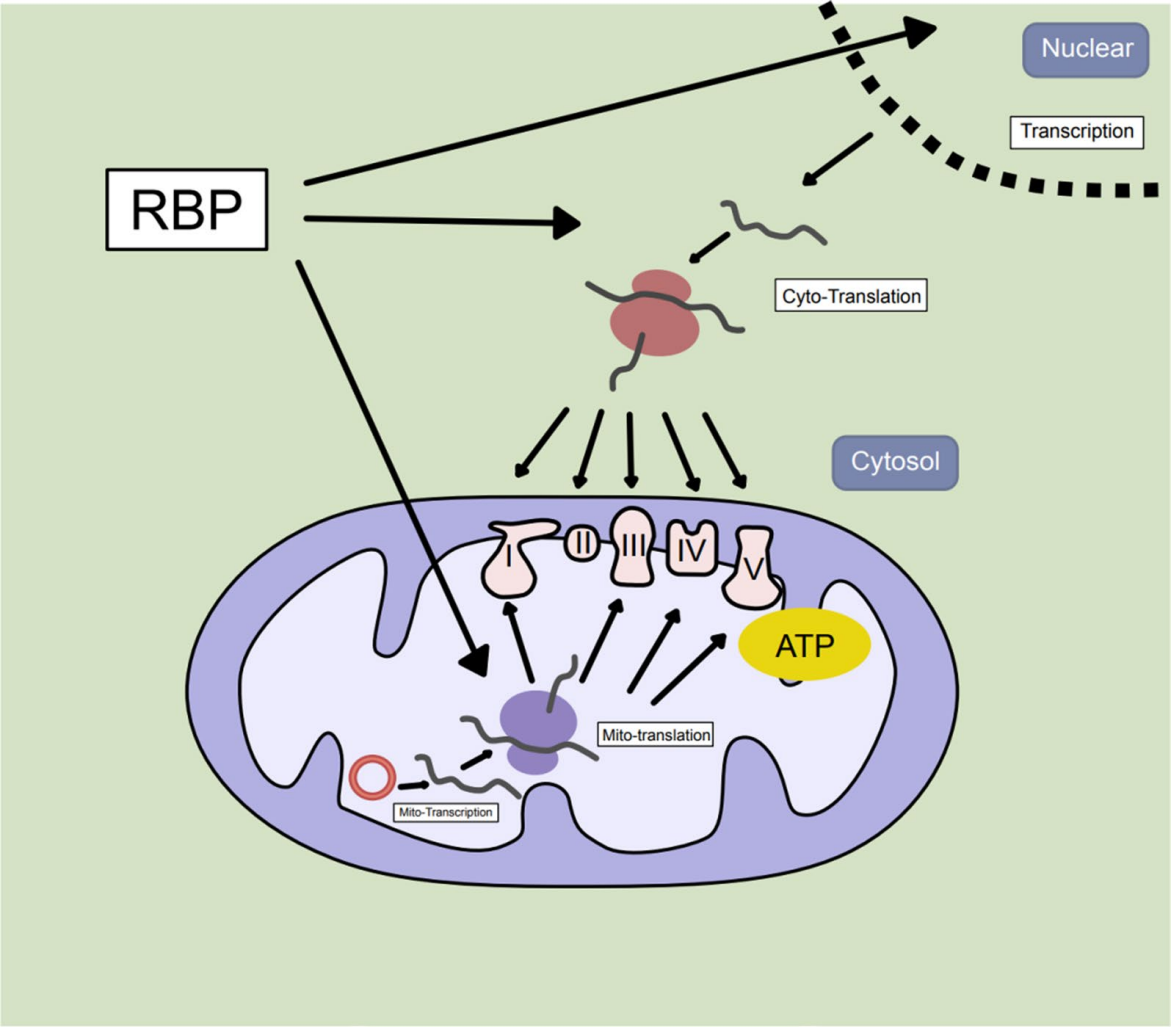
RNA-binding proteins participate in the translation of oxidative phosphorylation complex subunits. The oxidative phosphorylation complex is encoded by both the nuclear and mitochondrial genomes, and the balance of the two translation systems ensures energy production. RNA-binding proteins can participate in both cytoplasmic and mitochondrial protein translation

Changes in mRNA translation are both rapid and adaptive, and translational reprogramming is necessary for sustaining cancer cell proliferation. Deficiencies in translation caused by carcinogenic signaling pathways and microenvironmental stress lead to tumorigenesis, development, and drug resistance [19]. Initially, Ras and Akt signals were found to influence the tumor proteome in glioblastoma by regulating mRNA translation efficiency of proteins that regulate growth, transcriptional regulation, intercellular interactions and morphology [50]. Ribosome sequencing of malignant mesothelioma (MpM) revealed that mRNA translation, required for ribosome assembly and mitochondrial biogenesis, is selectively increased in MpM, resulting in increased mRNA translation, abnormal mitochondrial morphology and oxygen consumption, and metabolic reprogramming [50]. The inhibition of translation can reduce tumor growth and extend animal model survival. In mammalian eukaryotic cells, the majority of mRNA is transcribed in the nucleus before being transferred to the cytoplasm for translation and expression. Depending on intracellular and extracellular signals, tumor cells hijack the post-transcriptional mechanism and adjust the level of protein expression through the selective binding and translation regulation of RBP-RNA (Fig. 1).

### Regulation of cytoplasmic protein translation by RNA-binding proteins

RBPs participate in every stage of post-transcriptional regulation and are spatiotemporally specialized. The RNA binding domain’s adaptability and structural flexibility allow it to control the metabolism of a vast number of transcripts and the fate and function of each transcript inside the cell, resulting in the emergence and growth of tumors and the maintenance of cell homeostasis [51]. RBP is dysregulated in numerous cancers, affecting the expression and function of oncoproteins and tumor suppressors [52]. Through its binding to RNA’s 5’ or 3’ untranslated regions (UTR), RBP participates in translation with varying binding abilities, affecting the efficiency of translation of specific mRNAs. LARP1 is a target protein of mTORC1 that regulates the translation of 5’ TOP sequence mRNA. Despite some studies showing that LARP1 inhibits translation, LARP1 is also thought to promote the stability and translation of mRNAs that contain the 5’ TOP, such as ribosomal RNA [53]. Tumor cells require a large quantity of ribosomes to sustain their development and proliferation. The aberrant balance of ribosome biosynthesis may result in specific dysregulation of protein synthesis, which has become a hallmark of cancer cells [54, 55]. In addition, LARP1 can positively regulate the mRNA expression of multiple components of the mTOR carcinogenic pathway and other anti-apoptotic [B-cell lymphoma 2 (BCL2)] and pro-migration [Y-box binding protein-1 (YB1)] proteins, leading to LARP1-mediated cervical cancer tumorigenesis [56–58]. Human antigen R (HuR) is one of the most extensively studied RBPs in tumors and can enhance the stability of mRNA or promote the translation of target mRNA, such as BCL2, silent information regulator sirtuin 1 (SIRT1), and X-linked inhibitor of apoptosis (XIAP), ultimately leading to the increased proliferation, survival, and invasion capabilities of tumor cells [59].

By regulating mRNA stability or degradation, cytoplasmic RBP also affects cell survival and death through the respiratory chain complex. Clustered mitochondria homolog (CLUH) is an RNA-binding protein that binds specifically to the mRNA of the nuclear-encoded mitochondrial protein, maintains target mRNA stability, and prevents target degradation during translation [60]. When CLUH is absent, the target mRNA's attenuation increases and the encoded protein level decreases, resulting in aberrant respiratory chain complexes [61]. The iron deficiency-induced RNA-binding protein tristetraprolin (TTP) regulates gene expression by promoting the degradation of target mRNAs, including respiratory chain subunits encoding Fe/S clusters, such as NADH:ubiquinone oxidoreductase core subunit S1 (NDUFS1) in complex I and Ubiquinol cytochrome c reductase (UQCRFS1) in complex III [62]. In iron deprivation, these processes are unstable and prevent mitochondria from overusing iron, improving survival. The absence of Fe/S cluster complex III formation due to TTP deletion in mice results in iron deficiency. This iron deficiency, exacerbated by heart dysfunction, triggers the generation of reactive oxygen species, ultimately leading to cell death [62, 63]. Through its cold shock domain, the RNA-binding protein YB1 can block the translation of nuclear-encoded subunits NADH:ubiquinone oxidoreductase subunit A9 (NDUFA9), NADH:ubiquinone oxidoreductase subunit B8(NDUFB8), SDHB, and UQCRFS1 [64]. Engrailed and Lin28a initiate the translation of NDUFS1 and NADH:ubiquinone oxidoreductase subunit S3 (NDUFS3) and NADH:ubiquinone oxidoreductase subunit B3 (NDUFB3) and NDUFB8, respectively [65].

RBP can also bind to the mRNA of other mitochondrial function–related proteins to regulate mitochondrial function and homeostasis. CLUH interacts with ribosomal proteins, translation factors, and RNA-binding proteins GTPase-activating protein-(SH3 domain)-binding protein 1 (G3BP1) and GTPase-activating protein-(SH3 domain)-binding protein 2 (G3BP2), promotes mitochondrial co-translational import in the outer mitochondrial membrane, and affects the translation ability of mRNAs, including those involved in respiratory chain function, tricarboxylic acid (TCA) metabolism, fatty acid oxidation, and amino acid catabolism [66]. Additionally, CLUH regulates the PTEN-induced kinase 1 (PINK1)-parkin mitophagy pathway, which contributes to mitochondrial network remodeling. The cytoplasmic RBPs are important for maintaining, replicating, regulating transcription, and regulating the translation of mitochondrial genes [67]. According to research in *Drosophila*, the mitochondrial outer membrane protein MDI can attract Larp to the mitochondrial surface and promote the translation of the mitochondrial transcription factor (TFAM) and mitochondrial DNA polymerase, increasing the expression of mitochondrial genes and subtly altering the translation of mitochondria-encoded proteins [68].

### Regulation of mitochondrial protein translation by RNA-binding proteins

Mitochondrial and cytoplasmic translation are closely coordinated under different energy requirements. Mitochondrial mRNA translation is slower than cytoplasmic translation; thus, it is the rate-limiting step in effective OXPHOS-system ATP production. mtDNA translation must be rapidly adjusted in cells and tissues according to metabolism and energy demands [69]. All proteins apart from the 13 encoded by mitochondria are transcribed in the nucleus and translated in the cytoplasm, requiring cross-compartment processes. The transcription and translation of mtDNA proceeds in the mitochondrial matrix independently of the processes of nuclear DNA. Since mitochondrial genome promoters are relatively few, transcriptional regulators are insufficient to control gene expression quickly and accurately. Thus, mitochondria are highly dependent on RNA-binding proteins to regulate gene expression after transcription [70].

**Fig. 2:**
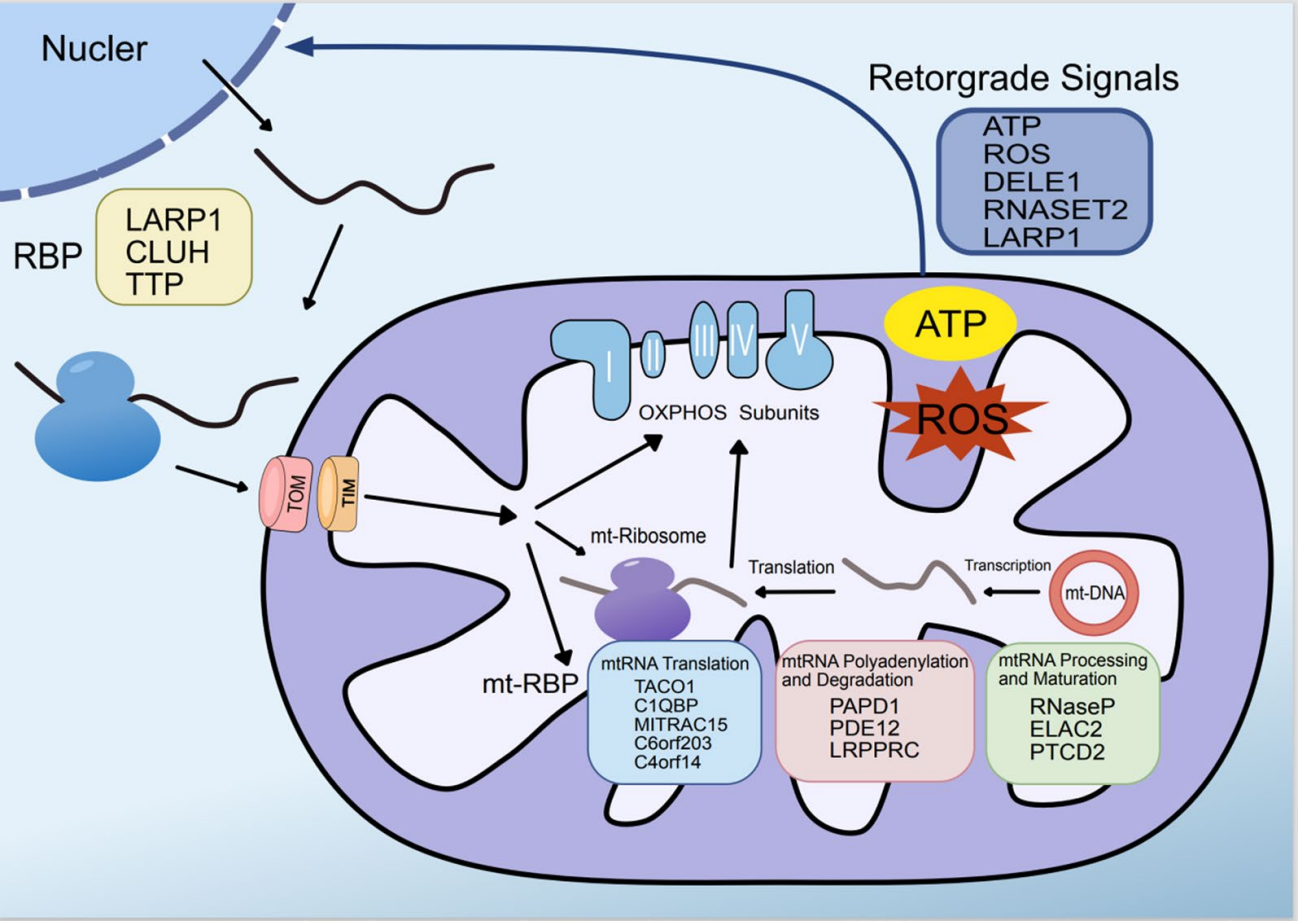
RNA-binding proteins participate in both cytoplasmic and mitochondrial protein translation. Subunits of the mitochondrial oxidative phosphorylation (OXPHOS) complex are encoded by both nuclear and mitochondrial genomes, and the balance of the two translation systems ensures energy production. Mitochondrial RNA-binding proteins, such as RNaseP, ELAC2, PTCD2, PAPD1, PDE12, LRPPRC, TACO1, C1QBP, and MITRAC15, are involved in mitochondrial RNA processing, polyadenylation, degradation, and mRNA translation. After translation in the cytoplasm, these proteins are transported into the mitochondria through TOM and TIM channels to exert their functions. In addition, mitochondrial retrograde signals, such as ATP, ROS, DELE1, RNASET2, and LARP1, can also regulate cytoplasmic protein translation

The mitochondrial transcriptome comprises polycistronic transcripts, suggesting that RNA-binding proteins regulate mitochondrial gene expression after transcription during RNA processing, maturation, translation, and decay. However, the steady-state abundance of each individual mRNA and tRNA differs substantially [71]. In the mt-RNA metabolism process, mitochondrial RBP (mt-RBP) plays a role in regulating gene expression after transcription, including the production of polycistronic transcripts, processing them into single transcripts, modifying, stabilizing, translating, and degrading mt-RNA [72] (Fig. 2). Overall, this process affects mitochondrial function and homeostasis through its effect on the mitochondrial respiratory chain subunits encoded by mitochondria. Cellular flexibility and the capacity of the oxidative phosphorylation complex to create ATP are governed by the regulation of translation by RNA-binding proteins [73]. By regulating mitochondrial encoded protein expression after transcription, mitochondrial RBP reshapes mitochondrial function, finely controls mitochondrial energy metabolism, and coordinates with other cellular processes quickly, flexibly, and powerfully. Dysfunction of the respiratory chain may cause cancer and other diseases due to mitochondrial gene expression defects [74]. By controlling these RBPs, cancer cells also sustain respiration and generate energy for environmental adaptation. To better understand mitochondrial function, we must first understand how tumor cells regulate mitochondrial translation through RBPs [74]. Such an understanding might open up new avenues for cancer treatment by furthering our conception of the strong flexibility of tumor cells in translational balance and energy regulation.

As soon as polycistronic transcripts are generated, mtRBPs take part in their processing and maturation: long polycistronic transcripts undergo Ribonuclease P (RNaseP) and elaC ribonucleaseZ2 (ELAC2) endonuclease processing to release individual transcripts [75, 76]. Knockout of RNaseP in HeLa cells increases the abundance of mitochondrial precursor transcripts and decreases the levels of mRNA, rRNA and tRNA, reducing mitochondrial translation, ribosome stability and respiration [77]. The mitochondrial RNaseP protein3 (MRPP3) enzyme is a component of the RNAseP complex and is essential for the processing of precursor RNA. In the absence of MRPP3, pre-RNA processing in mitochondria ceases, and mitochondrial translation decreases [78]. Knockdown of mitochondrial RNaseZ results in abnormal 3’ end processing of mitochondrial tRNA, accumulation of precursor transcripts and loss of mature tRNA. However, it has no significant effect on 5’ end processing of mitochondrial tRNA [79]. The pentapeptide repeat domain protein 2 (PTCD2) is a mitochondrial RNA-binding protein that transports mt-mRNA to mitochondrial ribosomes and affects the translation of MT-COX3 by participating in the 5' end processing of MT-CYB mRNA in HeLa cell lines. The deletion of the PTCD gene reduces the efficiency of MT-COX3 translation and impairs the activity of complex IV. Additionally, it influences the processing of the coding and noncoding regions of MT-ND5 and MT-CYB [80]. Thus, RNA-binding proteins participate in mitochondrial RNA processing and maturation to regulate mitochondrial translation.

In addition to MT-ND6 mRNA, all other mitochondria-encoded mRNAs undergo polyadenylation at the 3' end, which is an essential step in their development. The poly (A) tail is required for mitochondrial RNA expression. The removal of the poly (A) tail by introducing cytoplasmic poly (A) -specific 3’ 5’exonuclease (PARN) into mitochondria can increase the stability of some mRNAs or decrease the stability or have no impact on other mRNAs [81]. For instance, a decrease in poly (A) polymerase associated domain containing 1 (PAPD1) in mitochondria destroys the mRNA of MT-COX1, MT-COX2, MT-COX3, and MT-ATP8/MT-ATP6 but has no effect on the mRNA of MT-ND3, whereas 2’-phosphodiesterase phosphodiesterase 12 (PDE12) decreases the poly (A) tail length of MT-ND1 mRNA and increases its abundance [82]. Mitochondrial mRNA can exist in both adenosine and non-adenosine forms, and their roles vary. Both polyadenylation and deadenylation influence the body translation of linear granules and mRNA stability. The PDE12 protein is a deadenylation enzyme that influences a limited number of mitochondrial mRNA homeostasis levels and mitochondrial translation. It is unknown how the deadenylation of particular a mRNA impacts the translation of all mitochondrial-encoded proteins and whether other enzymes can deadenylate the poly (A) tail of the remaining mRNA [82]. The PAPD1 enzyme catalyzes poly (A) tail addition to 10 mRNAs in mammalian mitochondria, although its knockdown or mutation does not remove the oligoadenylation tails [83]. leucine-rich pentatricopeptide repeat-containing (LRPPRC) is overexpressed in several malignancies and is involved in RNA stabilization, processing, translation, and other activities [84]. The poly (A) tail length of mitochondrial mRNA is impacted by the loss of LRPPRC, which is required to preserve the untranslated polyadenylated mRNA that can stabilize the transcripts of MT-COX1, MT-COX2, and MT-COX3 [85]. LRPPRC knockout decreases mitochondrial mRNA levels and mitochondrial translation [86, 87]. Additionally, LRPPRC forms a complex with the mitochondrial RNA-binding protein SRA stem-loop-interacting RNA-binding protein (SLIRP) to stabilize mRNA molecules that do not bind to mitochondrial ribosomes, hence altering the translation of particular subsets of transcripts [88, 89]. These studies indicate RNA-binding proteins participate in mitochondrial polyadenylation and degradation to regulate mitochondrial translation.

Mitochondrial mRNA translation takes place in the mitochondrial matrix and is conducted by mitochondrial ribosomes. The primary process comprises four steps: initiation, extension, termination, and recycling of ribosomes. Some RBPs can participate in mitochondrial translation and selectively regulate the translation of specific mRNAs by binding to them. Cytochrome c oxidase deficiency has been identified in patients with late-onset Leigh syndrome; however, their MT-COX1 mRNA levels were normal, showing that translation activator of cytochrome c oxidase 1 (TACO1) can bind to MT-COX1 mRNA and initiate its translation [90, 91]. Richman et al. discovered that TACO1 binds specifically to the MT-COX1 protein and promotes translation by facilitating its attachment to mitochondrial ribosomes. The MT-COX1 protein is significantly reduced in TACO1 mutant mice [92]. The RNA-binding activity of Complement C1q binding protein [C1QBP (p32)], a protein found in the mitochondrial matrix, may be crucial for the proper and efficient translation of mtDNA. C1QBP can bind to all mitochondrial mRNAs, and while it only directs mRNA to the mitochondrial ribosome, it considerably aids efficient initiation and/or elongation events. The absence of p32 results in severe abnormalities in mitochondrial translation [93]. Cytochrome c oxidase 15 (MITRAC15) can promote the translation of MT-ND2 in the MT-ND2 ribosome-neogenesis complex [94], which suggests that RNA binding protein controls mRNA and affects the plasticity of mitochondrial protein translation.

The mitochondrial genome encodes only two rRNAs for mammalian mitochondrial ribosomes: the small subunit 12SrRNA and the large subunit 16SrRNA. Some mitochondrial ribosomal RNA-binding proteins may significantly impact the translation and recognition of mitochondrial mRNA and the translation of mitochondrial proteins. C6orf203 is essential for the maturation and regulation of mitochondrial large subunit activity in human cells and interacts with mitochondrial ribosomal large subunit (mt-LSU). Absence of C6orf203 reduces mitochondrial translation, resulting in respiratory failure; therefore, C6orf203 is an efficient regulator of mitochondrial translation [95, 96]. C4orf14 [Nitric oxide-associated protein 1 (NOA1)] contains an RNA-binding domain that supports the assembly of the mitochondrial 28S small subunit, which is required for optimal mitochondrial translation and respiratory activity [97, 98]. These results provide conclusive evidence for the crucial involvement of mitochondrial RBP in mitochondrial RNA metabolism and, consequently, in the proper translation of mitochondrial proteins (Fig. 2).

### The role of non-coding RNA in coordinating nuclear-mitochondrial function by RNA-binding proteins

Post-transcriptional regulation of mRNAs can also occur through the activity of diverse non-coding RNAs, including microRNA (miRNA), long non-coding RNA (lncRNA), and circular RNA (circRNA) [99]. Increasing numbers of studies have provided evidence that ncRNAs act as crucial molecules in the initiation and progression of the tumor by their involvement in transcriptional and post-transcriptional regulation processing. RBP and lncRNA are dysregulated in a variety of human cancers, and it can change the fate and function of lncRNAs by regulating their stability, transport and transcription and further playing a role in tumorigenesis and development [100]. For example, insulin-like growth factor 2 mRNA-binding protein 1 (IGF2BP1) binds to LINC01093,, which interrupts the interaction of IGF2BP1 with GLI1 mRNA, resulting in degradation of GLI1 mRNA and the subsequent regression of HCC [101]. HuR can bind to LncRNA-HGBC to promote its stability, thereby promoting the development and metastasis of gallbladder cancer (GBC) [102]. circRNAs play important regulatory roles in cancer development [103]. Mechanistically, circTHBS1 behaves as a miR-204-5p sponge to enhance the INHBA expression, and it also stabilizes the INHBA mRNA mediated by HuR, consequently activating the TGF-beta pathway [104].

RNA binding proteins may regulate the subcellular localization of ncRNA, that is, compartmentalization changes affect its function. LncRNA-RMRP can change the compartmental changes of lncRNA-RMRP by binding to RBP HuR and GRSF1, respectively, so as to locate in the cytoplasm and mitochondrial matrix. Loss of GRSF1 lowered the mitochondrial levels of RMRP, in turn suppressing oxygen consumption rates and modestly reducing mitochondrial DNA replication priming [105]. RNA binding protein AUF1 regulates mitochondrial function by regulating the stability of lncRNA RPPH1 and promoting its mitochondrial localization [106]. The RNA-binding protein RALY promotes post-transcriptional processing of specific subgroups of miRNAs (miR-483, miR-676, and miR-877), down-regulates the respiratory chain subunits ATP5I, ATP5G1, ATP5G3, and CYC1 in mitochondria, thereby reprogramming mitochondrial metabolism in cancer cells to promote colorectal cancer (CRC) progression [107]. RNA-binding protein Musashi RNA binding protein 2 (MSI2) might be responsible for the distribution of miR-301a-3p between cytosol and mitochondria in endothelial cells [108]. These compartmental changes may be the way in which RBP regulates the interaction between nucleus and mitochondria, thereby regulating mitochondrial gene expression and affecting mitochondrial function. This suggests that the regulation of ncRNA by RBP may be involved in the development of tumors.

### Relationship of nuclear–mitochondrial anterograde signals to the mitochondrial gene expression

The stoichiometric assembly of nuclear- and mitochondrial-encoded proteins is necessary for the oxidative phosphorylation system. Studies have revealed a strong correlation between nuclear and mitochondrial gene expression and communication between mitochondrial and cytoplasmic translation. The imbalance of these two translation systems affects some pathophysiological processes [109]. The correlation between mitochondrial and cytoplasmic ribosomal proteins in mouse population genetics indicates a translation balance. Inhibiting mitochondrial translation can initiate the activating transcription factor ATF4/ATF5-dependent response and coordinate cytoplasmic translation to prolong life [110]. Mitochondrial ribosome imprinting analysis of 5 cell types revealed that the translation level of encoded proteins accurately corresponded to the cellular level of the entire OXPHOS complex. Mitochondrial translation rates were related to the relative abundance of complexes, and the lack of mitochondrial translation produced an imbalance between cytoplasmic translation and mitochondrial translation, resulting in protein toxicity [30].

It was discovered in *Saccharomyces cerevisiae* mitochondria that increasing the translation of nuclear-encoded respiratory chain subunits can stimulate the translation of mitochondrial coding subunits, indicating that cytoplasmic translation and mitochondrial translation are synchronized [111]. Although the specific molecular mechanism of the synchronization of cytoplasmic and mitochondrial translation remains unknown, there exists a coordination mechanism that promotes translation equilibrium. Synergistic regulation might regulate mitochondrial translation [17]. The process of translation synchronization occurs over a longer period of time in eukaryotic cells, and the mitochondrial translation device must detect the arrival and assembly of nuclear-encoded respiratory components and modify the rate of mitochondrial translation as necessary. The assembly factors C12orf62, Cytochrome c oxidase 12 (MITRAC12), and MITRAC15 regulate the synthesis of mitochondrial-encoded MT-COX1 and MT-ND2 by introducing nuclear-encoded peptides [112]. Ricarda et al. showed that human mitochondrial ribosomes govern the plasticity of mitochondrial translation based on the input of nuclear-encoded subunits, and ribosomes that express mitochondrial-encoded MT-COX1 mRNA selectively bind to cytochrome c oxidase assembly components in the intima. The faulty assembly of cytochrome c oxidase inhibits mitochondrial translation in the ribosome nascent chain complex, accompanied by MT-COX1 translation products that are partially membrane-inserted [113]. This complex describes the translation product's beginning state and can be obtained for assembly. These results show a mammalian translation plasticity route in mitochondria, permitting the adaptation of mitochondrial protein synthesis to the input of nuclear-coding subunits [113].

Since mitochondrial translation requires the coordination of two translation systems, ribosomes, mitochondrial translation initiation, elongation-related factors, mitochondrial RNA processing, and translation-related factors are translated in the cytoplasm [76, 114]. Several mitochondrial ribosomal subunits are abnormally expressed in many cancers, which is associated with poor prognosis and cancer invasiveness. An investigation using high-throughput sequencing and analytics tools showed that mitochondrial ribosomes are closely associated with cancerous tumors [115]. The factors affecting mitochondrial protein translation can be used as biomarkers to track specific tumor molecular functions. Genome-wide association studies (GWASs) and cancer proteome analyses have demonstrated that mitochondrial ribosomal subunit composition and function lead to tumorigenesis [31]. Over 95 genes associated with mitochondrial biogenesis and mitochondrial translation were found to be considerably upregulated in breast cancer cells relative to the surrounding matrix, including approximately 40 mitochondrial ribosomal proteins that functionally influenced mitochondrial translation [116]. Studies of head and neck squamous cell carcinoma have shown that decreased mitochondrial ribosomal subunit MRPL11 leads to decreased MT-COX2 expression [117]. Mitochondrial assembly factors regulate mitochondrial translation, and their role in tumor growth and development is well established. The mitochondrial Tu translation elongation factor (TUFM) affects mitochondrial translation, altering mitochondrial respiratory chain function. High TUFM expression in gastrointestinal stromal tumors (GISTs) is associated with tumor occurrence, progression, and prognosis [118]. Overexpression of mtEF4 may cause mitochondrial translation disorders and cancer development [40]. Knockdown of mtEF4 activates the mTOR signaling pathway and leads to the activation of cytoplasmic protein translation, indicating that mTOR signaling is a key compensation method for mitochondrial translation defects [119]. The MT-COX1 chaperone C-X9-C motif containing 1 (CMC1) was found to enhance the stability of newly synthesized MT-COX1 in HEK293T cells [120]. The communication from nucleus to mitochondria ensures the normal function of mitochondria, which may be through direct regulation of the expression of nuclear encoded OXPHOS subunits or indirect regulation of factors involved in mtDNA transcription, translation and thus affect the expression of mitochondrially encoded OXPHOS subunits. The positive regulation is a prerequisite for mitochondrial energy production.

### The effect of mitochondrial-nuclear retrograde signals on protein translation

Bidirectional communication between mitochondria and the nucleus is required to coordinate the expression, translation and assembly of mitochondrial OXPHOS complexes encoded by mitochondria and nuclear genomes to ensure optimal mitochondrial function. Mitochondrial function is mediated by nuclear-encoded genes through anterograde (nuclear-to-mitochondrial) signaling, ensuring that local translation of the mitochondrial outer membrane is coordinated with mitochondrial protein input and OXPHOS complex assembly and that mitochondrial ribosomes respond to nuclear-encoded subunits (Fig. 2). Input-regulated translation plasticity meets cellular needs. Inhibition of cytoplasmic translation affects mitochondrial function in various ways, leading to two-way mitochondrial transmembrane protein homeostasis signals [121]. The process of cell solute protein homeostasis, nuclear stress signal transduction and mitochondrial translation accuracy is closely coordinated and determines the life span of cells [122]. Additionally, mitochondria can act as a signal sensor to respond to changes, such as protein homeostasis pressure, insufficient energy and increased reactive oxygen species (ROS) production, and transmit signals to the nucleus, triggering transcriptional reprogramming for metabolic adaptation and ultimately changing nuclear gene expression. This process is referred to as retrograde signaling [123]. Thus, communication between mitochondria and the nucleus provides cells with a dynamic regulatory network that enables them to respond quickly to changing environments.

Retrograde signals can act as ‘informants’ for governing protein translation. When cells face external stress, mitochondria act as sensors. Retrograde signals seem to affect a wide range of processes in cancer progression, including the activation of signaling pathways that regulate metabolic adaptation, antioxidant systems, cell proliferation, apoptosis resistance, chemical resistance, and cell migration and invasion. Changing mitochondrial function regulates adaptive changes in nuclear gene expression and metabolism mediated by specific transcription factors. Some small molecules can transmit information as retrograde signals; for example, ROS, NAD^+^/NADH ratio, acetyl CoA, ATP, and Ca^2+^ [124, 125]. ROS overflowing from mitochondria can activate the kinase general control nonderepressible 2 (GCN2), thereby phosphorylating eIF2α and inhibiting the translation of cytoplasmic proteins [126]. RNA binding proteins may act as a reverse signal. Mitochondrial membrane gap ribonuclease T2 (RNASET2) can degrade mitochondrial RNA and rRNA on the outer membrane of mitochondria in the cytoplasm and may regulate the balance mechanism of gene expression inside and outside mitochondria through feedback degradation activity [127].

Some proteins can also act as alarms when their localization changes. Dysfunctional mitochondria may activate the mitochondrial stress response. For example, when there are too many misfolded proteins in the mitochondrial species, the mitochondrial unfolded protein response can be activated, and the ATF5 originally transported to the mitochondrial species degraded by mitochondrial enzymes will enter the nucleus to regulate the transcription process, thereby promoting the transcription of genes related to mitochondrial protein homeostasis [128]. Under stress, the C-terminal fragment of DAP3-binding cell death enhancer 1 (DELE1) located in mitochondria will transfer from the mitochondria to the cytoplasm, bind and activate kinase HRI, phosphorylate eIF2α, trigger the integrated stress response (ISR), and regulate the translation of cytoplasmic proteins [129]. The localization changes of some RNA binding proteins may be used as reverse signals. Upon binding to mitochondria-related mRNA, LARP1 may promote co-translation into mitochondria when found in the outer membrane of mitochondria. LARP1 can enter stress granules when exposed to stress, resulting in translation stagnation and affecting mitochondrial function. In addition to positive regulation, reverse regulation ensures the metabolic capacity of mitochondria and the environmental adaptability of tumor cells by sensing small molecule signals, protein localization changes, and timely fine-tuning gene expression.

### The role of liquid–liquid phase separation in mitochondrial-cytosolic translational balance

Liquid–liquid phase separation (LLPS) is a dynamic, reversible, membrane-free aggregation structure that is highly concentrated in RNA binding protein (RBP) and RNA. LLPS is created by weak multivalent interactions between proteins and nucleic acids [130, 131]. These RBPs have intrinsically disordered and unstructured RNA-binding domains that impact local RNA molecular concentration and aggregation and are crucial for mRNA metabolism. When eukaryotic cells are stimulated or tampered with, mature mRNA cannot be quickly translated into proteins. These momentarily untranslated or translated messenger ribonucleoproteins (mRNAs) then polymerize with RNA-binding protein (RBP) to create membrane-free messenger ribonucleoprotein (mRNP) particles [132]. These granules include nuclear granules, such as Cajal bodies [133], side spots [134], and nucleoli [135], cytoplasmic granule, such as stress granules [136] and P bodies [137], and mitochondrial RNA granules (MRG) in mitochondria. Numerous studies have demonstrated that aberrant LLPS can influence the development and progression of cancer by raising the concentration of particular proteins or RNA in the particles that govern biological processes [138, 139]. Additionally, LLPS can function as a stress sensor. When environmental stress conditions such as heat stress or pH change, these granules can be produced and regulated promptly as a tumor survival strategy [140].

mRNA translation and turnover are at the core of gene expression regulation. In eukaryotes, RNP particles isolate mRNA and RNA-binding proteins in response to changing cellular needs and physiological conditions. mRNA’s fate, such as transport, degradation, or translation, is determined by the composition of mRNP. mRNA translation can be determined through mRNP remodeling by regulating its protein composition [141]. mRNP particles significantly impact mRNA function and cell signal transduction and are also intimately associated with diseases. It is known that Stress granules contain translation-silenced mRNAs and that mRNAs related to active ribosome translation are in dynamic balance, thereby regulating protein translation [142]. The study of mitochondrial RNA particles can provide new ideas for regulating mitochondrial mRNA translation; however, additional evidence remains to be obtained. Here, we mainly describe the regulation of SG and MRG on translation in tumors.

### Stress granules

**Fig. 3:**
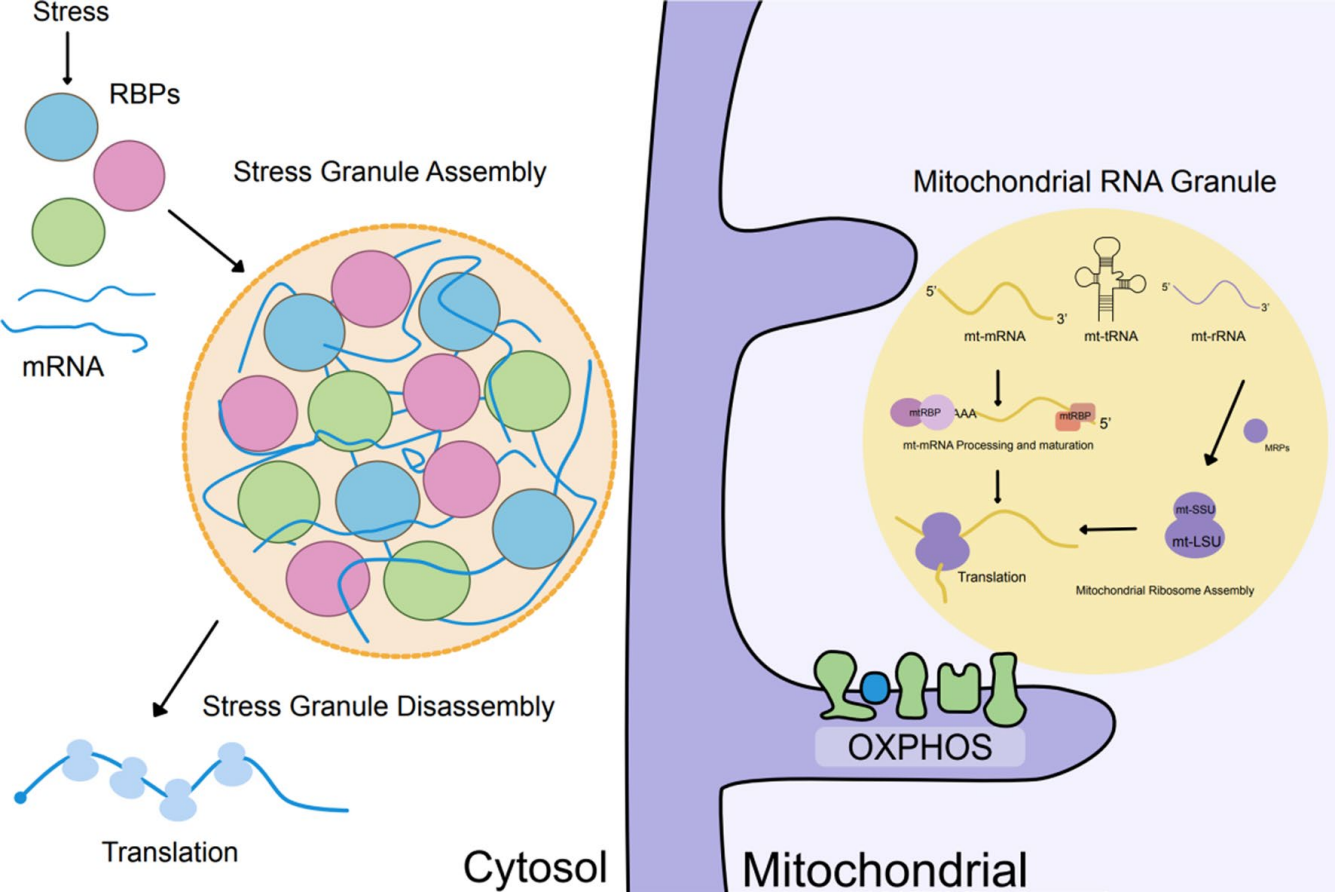
Stress granules and mitochondrial RNA granules. During stress, RNA-binding proteins bind to stalled mRNAs and form stress granules through liquid–liquid phase separation. When stress subsides, stress granules disassemble and translation resumes. In mitochondria, transcriptionally active mitochondrial DNA is processed and matured within mitochondrial RNA granules, while mitochondrial ribosomes are assembled within mitochondrial RNA granules. After co-translational insertion into the inner membrane, mitochondrial mRNAs are degraded

Stress granules (SG) are among the most researched LLPSs; SGs are membraneless dynamic granules formed by translationally stagnant mRNA and RBP [141] (Fig. 3). When cells are exposed to heat shock, hypoxia, chemical stimulation, viral infection, oxidative stress, and aging, eukaryotic initiation factor 2α (eIF2α) can become phosphorylate, inhibiting its guanylate conversion function and the formation of the ternary initiation complex, preventing translation [143, 144]. Additionally, SG production can occur independently of eIF2 phosphorylation. For instance, interfering with the binding of eIF4E to eIF4G or decreasing the activity of eIF4A can deactivate the eukaryotic translation initiation factor 4 complex (eIF4F) and halt translation initiation [145]. These mRNAs are condensed by RNA binding protein (RBP) and form stress granules with ribosome components and translation initiation factors.

Typically, cancer cells are characterized by hypoxia, nutritional deprivation, and elevated osmotic pressure. Under challenging circumstances, stress granule formation is an adaptive regulatory mechanism that protects tumor cells from apoptosis. SG creation and dynamics can alter the location, translation, and degradation of messenger RNA and change signaling pathways [146, 147]. In vivo, tumor cells are resistant to stress and maintain translation balance by producing SGs, which inhibit apoptosis and promote drug resistance. The inhibition of hypoxia-induced stress granule formation can increase HeLa cells' drug sensitivity [148]. The non-SG-forming breast cancer cell line Hs587T is more sensitive to bortezomib than other SG-forming cell lines [149]. SGs can be mobilized by tumor microenvironment factors, resulting in enhanced adaptability and survival of tumors under stressful settings. SGs can temporarily store mRNA [150], safeguarding it from degradative processes and reactivating translation when the stress subsides. SGs can assist cells in adapting to the local demand for quick production of cytoprotective proteins to maintain vitality. Some of these undesirable transcripts are identified, briefly suppressed, and stabilized, which is vital for the survival of tumor cells. Stress granule disasembly is accompanied by the recovery of a significant level of protein synthesis and single mRNA translation processes during recovery from stress [151]. Some RNA-binding proteins stimulate the disintegration of stress granules. Staufen can stabilize the binding of mRNA to ribosomes; knocking it down enhances stress granule production, whereas overexpression inhibits stress granule formation [152]. The phosphorylation of Grb7 by focal adhesion kinase (FAK) weakens the interaction with other stress granule components [such as HuR and T-cell restricted intracellular antigen-1 (TIA-1)] and the binding to specific mRNAs. Impaired phosphorylation of growth factor receptor-bound protein-7 gene (Grb7) by FAK renders stress granules incapable of decomposing and restoring translation. SGs can also selectively isolate important signal transduction pathway components [153], such as receptor for activated C kinase 1 (RACK1) (p38/JNK pathway), TNF receptor associated factor 2 (TRAF2) (NF-кB route), Raptor (mTOR pathway), and RhoA/ROCK (Wnt pathway), thus interrupting the complete downstream effector cascade. This can result in cell abnormalities, which can eventually lead to various diseases. Multiple SG-forming molecular components are associated with carcinogenesis and tumor-related signaling pathways. SGs are variable and dynamic and recruit many RNA-binding and other proteins. Stress granules have different functions depending on the protein composition. For example, zinc finger AN1-type containing 1 (ZFAND1) within the stress granules can attract 26 s and valosin containing protein (VCP) to aid in the clearance of the granules, while 70 kDa heat shock protein (HSP70) can halt the aggregation of improperly folded proteins within SGs to create SGs with phase transition [154].

Stress granules selectively preserve mitochondria-related mRNAs to ensure mitochondrial function. The RNA-binding protein CLUH ensures the stability and translation of transcripts involved in mitochondrial catabolism (fatty acid oxidation and amino acid degradation) and ketogenesis and inhibits mTORC1-dependent translation of mRNAs involved in mitochondrial protein synthesis, thus reducing energy-consuming synthetic metabolism processes [61]. In addition, stress granules can regulate mitochondrial permeability [155]. Furthermore, mitochondrial stress conditions can induce the formation of cytoplasmic stress granules. Treatment with the respiratory chain inhibitor, succinate, leads to SG formation and translation inhibition [156]. Together, these findings suggest that stress granules may serve as a means of regulating cytoplasmic and mitochondrial translation.

### Mitochondrial RNA granules

Under physiological and stress conditions, LLPS plays a central role in mRNA metabolism and exhibits a variety of forms and functions. Most studies on RNA particles focus on the nucleus and cytoplasm. Recent studies have shown that RNA particles can also be found in mitochondria [157]; however, this is not due to stress (Fig. 3). The importance of LLPS in the mitochondria of tumor cells remains unclear. MRGs are membrane-free dynamic structures within mitochondria that provide a platform for various temporal and spatial regulation mechanisms for mitochondrial gene expression, such as RNA processing, maturation, ribosome assembly, and translation [158].

Various nuclear-encoded cofactors are involved in regulating mitochondrial genome expression, ensuring normal OXPHOS complex function. Proteins involved in mitochondrial genome translation are not randomly distributed in mitochondria but aggregate in a dotted pattern. A dot-like structure marked with 5-bromopyridine (Bru) was found for the first time in T-24 bladder cancer cells. In mitochondria, four members of the Fas-activated serine/threonine kinase (FASTK) family and G-rich sequence factor 1 (GRFS1) form discrete foci that co-localize with Bru-labeled dots [159, 160]. Within the mitochondrial matrix, newly synthesized RNA, RNA processing proteins, and mitochondrial nucleoid assembly factors form punctate compartments known as mitochondrial RNA granules (MRGs). Using live-cell super-resolution structured illumination microscopy and fluorescence recovery after photobleaching experiments, Timo et al. discovered that MRGs could rapidly exchange components and undergo fusion, exhibiting liquid-like properties. Fifty proteins were suggested to localize to MRGs with temporal or spatial specificity due to their ability to co-localize with BRU or MRG marker proteins GRSF1 and FASTK2 and their proximity to the nucleoid [161].

Different protein compositions within MRGs can form granules with different functions, serving as functional platforms for mtRNA metabolism at different stages [162]. Timo et al.'s research indicates that MRGs can rapidly exchange components and fuse, allowing for the regulation of the positioning of these granules to maintain proper assembly of the respiratory chain and oxidative phosphorylation function [163]. Enzymes involved in mtRNA processing, such as GRSF1, RNaseP subunits, and mitochondrial poly (A) polymerase (mtPAP), can act as processing platforms for mitochondrial RNA [164]. GRSF1, a soluble mitochondrial protein that localizes to RNA granules, coordinates the transcriptional storage and processing of mitochondrial mRNAs and long non-coding RNAs (lncRNAs) [165]. GRSF1 preferentially binds to specific RNAs transcribed from the mtDNA light chain promoter, including one mRNA and two lncRNAs containing binding sequences with AGGGD. Deletion of GRSF1 leads to the instability of several mRNAs and rRNAs, misloading of RNA on ribosomes, abnormal ribosome biosynthesis, and dysregulated synthesis of mitochondrial proteins [158]. MRGs containing enzymes involved in post-transcriptional nucleotide modifications of mtRNAs, such as mitochondrial rRNA methyltransferase 2 (MRM2), mitochondrial rRNA methyltransferase 3 (MRM3), mitochondrial transcription factor B1 (TFB1M), pseudouridylate synthase 1 (PUS1), and tRNA methyltransferase 10C, mitochondrial RNase P subunit (TRMT10C), can serve as centers for mtRNA maturation. MRGs also contain mitochondrial ribosome assembly factors, such as mitochondrial transcription termination factor 3 (MTERF3)/mitochondrial transcription termination factor (MTERFD1), Era like 12S mitochondrial rRNA chaperone 1 (ERAL1), DEAD-box helicase 28 (DDX28), and FASTK family proteins, as well as complete mitochondrial ribosome components, including mt-rRNA and mitochondrial ribosome proteins [160, 165–169], serving as a regulatory platform for mitochondrial translation. Additionally, mtDNA replication factors Twinkle and mitochondrial single-stranded binding protein (mtSSB) have been identified in MRGs, and their functions in granule formation and mtRNA processing/degradation have been determined [170]. Depletion of Twinkle can significantly reduce RNA retention time but does significantly affect RNA processing, which can affect mitochondrial translation, thereby altering mitochondrial function and homeostasis.

## Conclusions and future perspectives

When cells face different stimuli, translation control is an important mechanism for dynamically regulating gene expression, which can change the cell phenotype. The plasticity of mRNA translation is one of the key factors in tumor formation and development. Because mitochondria play an indispensable role in the occurrence, development, metastasis and drug resistance of tumors, mitochondrial protein translation is essential for mitochondrial oxidative phosphorylation, cell energy supply and other mitochondrial functions. Although the studies in the past decade have found that nuclear-mitochondrial communication plays a coordinating role in the transcription, translation and assembly of mitochondrial-encoded proteins and their adaptation mechanisms. With the deepening of research, it has been found that in addition to the regulation mechanism of compartmentalization originally possessed by cells such as nucleus and mitochondria, the regulation of new or temporary compartmentalization such as liquid–liquid phase separation, so that tumor cells can quickly respond to various stress conditions and produce appropriate responses, thereby further regulating the strong adaptability of tumors. At present, it is believed that in addition to the process of cell transcription and translation, the regulatory role of RNA-binding proteins in new or temporary compartmentalization such as liquid–liquid phase separation of stress granules and p-bodies will provide a new theoretical basis for explaining the role of mitochondrial protein translation in mitochondrial oxidative phosphorylation, cell energy supply and other mitochondrial function biogenesis, which deserves great attention.

So far, the establishment of a variety of sequencing and big data analysis methods can provide a more comprehensive understanding of the regulatory mechanism of RNA-binding proteins on mitochondrial translation. However, due to the extremely complex regulatory mechanism of mitochondrial translation, there are still many problems to be considered and studied. For example, in terms of time, the order of the role of RNA binding proteins in the separation of nucleus and liquid phase? Which cell signal transduction is involved in the transmission of information between the nucleus and mitochondria, between the nucleus or mitochondria and liquid–liquid phase separation? Are there still some unidentified RNA-binding proteins that directly regulate mtRNA to regulate mitochondrial translation at the mitochondrial level? How does RNA binding protein indirectly regulate the transcription or translation of mitochondrial DNA-encoded OXPHOS complex? In the case of tumor cell stress, RNA binding protein (RBP) selectively regulates the translation of mRNA or forms ribonucleoprotein granules in the form of liquid–liquid phase separation, thereby selectively regulating the proteome of cells to maintain cell homeostasis and regulate the strong adaptability of tumor cells. Does mitochondrial RNA binding protein bind to mitochondrial RNA to produce mitochondrial stress granules? In particular, the recovery of mitochondrial-related stress granule disassembly and translation in tumor cells and the maintenance of mitochondrial function in tumor cells play a role in the occurrence, development and metastasis of tumors and drug resistance need further study. In summary, through the role of RNA-binding proteins, it will further clarify the powerful co-ordination and decision-making role of mitochondria in tumorigenesis and development, and provide more innovative ideas for clarifying cells, especially tumor cells, to ensure mitochondrial function and improve cell adaptability. It can also be used as an effective cancer treatment approach and a strategy to overcome drug resistance.

## Supplementary Information

**Additional file 1.** The similarities and differences between cytoplasmic protein translation and mitochondrial protein translation, as well as their translation processes.

## Data Availability

Not applicable.

## Abbreviations

nDNA: Nuclear DNA
mtDNA: Mitochondrial DNA
OXPHOS: Oxidative phosphorylation
RBP: RNA-binding protein
mRNA: Messenger RNA
ATP: Adenosine triphosphate
PI3K: Phosphoinositide 3-kinase
mTOR: Mechanistic target of rapamycin
LARP1: La-related protein 1
rRNAs: Ribosomal RNAs
tRNAs: Transfer RNAs
MT-ND: Mitochondrially encoded NADH dehydrogenase subunit
MT-CYB: Mitochondrially encoded cytochrome b
MT-COX: Mitochondrially encoded Cytochrome c oxidase
MT-ATP: Mitochondrially encoded ATP synthase
ETC: Electron transport chain
Q/D: Quinupristin/dalfopristin
AMPK: AMP-activated protein kinase
MRPs: Mitochondrial ribosomal proteins
mtEF4: Mitochondrial translation elongation factor 4
MTIF2: Mitochondrial translation initiation factor 2 gene
MRPL: Mitochondrial ribosomal protein L
MRPS: Mitochondrial ribosomal protein S
MpM: Malignant mesothelioma
UTR: Untranslated regions
BCL2: B-cell lymphoma 2
YB1: Y-box binding protein-1
HuR: Human antigen R
SIRT1: Silent information regulator sirtuin 1
XIAP: X-linked inhibitor of apoptosis
CLUH: Clustered mitochondria homolog
TTP: Tristetraprolin
NDUF: Ubiquinone oxidoreductase core subunit
UQCRF: Ubiquinol cytochrome c reductase
SDHB: Succinate dehydrogenase complex iron sulfur subunit B
G3BP: GTPase-activating protein-(SH3 domain)-binding protein
TCA: Tricarboxylic acid
PINK1: PTEN-induced kinase 1
mt-RBP: Mitochondrial RBP
RNaseP: Ribonuclease P
ELAC2: ElaC ribonuclease Z 2
MRPP3: Mitochondrial RNase P protein 3
PTCD2: Pentapeptide repeat domain protein 2
PARN: Poly(A)-specific ribonuclease
PAPD1: Poly(A) polymerase associated domain containing 1
PDE12: Phosphodiesterase 12
LRPPRC: Leucine-rich pentatricopeptide repeat-containing
SLIRP: SRA stem-loop-interacting RNA-binding protein
TACO1: Translation activator of cytochrome c oxidase 1
C1QBP: Complement C1q binding protein
MITRAC15: Cytochrome c oxidase 15’
SrRNA: Small subunit ribosomal RNA
mt-LSU: Mitochondrial ribosomal large subunit
NOA1: Nitric oxide-associated protein 1
miRNA: MicroRNA
lncRNA: Long non-coding RNA
circRNA: Circular RNA
IGF2BP1: Insulin-like growth factor 2 mRNA-binding protein 1
GBC: Gallbladder cancer
CRC: Colorectal cancer
MSI2: Musashi RNA binding protein 2
ATF: Activating transcription factor
MITRAC12: Cytochrome c oxidase 12
GWASs: Genome-wide association studies
TUFM: Mitochondrial Tu translation elongation factor
GISTs: Gastrointestinal stromal tumors
CMC1: C-X9-C motif containing 1
ROS: Reactive oxygen species
GCN2: General control nonderepressible 2
RNASET2: Ribonuclease T2
DELE1: DAP3-binding cell death enhancer 1
ISR: Integrated stress response
LLPS: Liquid–liquid phase separation
mRNP: Messenger ribonucleoprotein
MRGs: Mitochondrial RNA granules
SGs: Stress granules
eIF4F: Eukaryotic translation initiation factor 4 complex
FAK: Focal adhesion kinase
TIA-1: T-cell restricted intracellular antigen-1
Grb7: Growth factor receptor-bound protein-7 gene
RACK1: Receptor for activated C kinase 1
JNK: C-Jun N-terminal kinase
TRAF2: TNF receptor associated factor 2
NF-кB: Nuclear factor kappa B
RhoA: RAGE/Ras homolog gene family member A
ROCK: Rho-associated coiled-coil kinase
ZFAND1: Zinc finger AN1-type containing 1
VCP: Valosin containing protein
HSP70: 70 KDa heat shock protein
FASTK: Fas-activated serine/threonine kinase
GRFS1: G-rich sequence factor 1
mtPAP: Mitochondrial poly (A) polymerase
lncRNAs: Long non-coding RNAs
MRM: Mitochondrial rRNA methyltransferase
PUS1: Pseudouridylate synthase 1
TRMT10C: TRNA methyltransferase 10C
ERAL1: Era like 12S mitochondrial rRNA chaperone 1
DDX28: DEAD-box helicase 28
mtSSB: Mitochondrial single-stranded binding protein
