# Supplementary material for: RNA binding protein: coordinated expression between the nuclear and mitochondrial genomes in tumors
Source: J Transl Med. 2023 Jul 28;21:512. doi: 10.1186/s12967-023-04373-3 (PMC10386658; doi:10.1186/s12967-023-04373-3)
Supplement: Supplementary file 1 — Additional file 1. The similarities and differences between cytoplasmic protein translation and mitochondrial protein translation, as well as their translation processes. [file 12967_2023_4373_MOESM1_ESM.docx]

**RNA binding protein:** **coordinated expression between the nuclear and mitochondrial genomes in tumors**

**Jiaoyan Ma^1^, Liankun Sun^1^, Weinan Gao^1^, Yang Li^1^ and Delu Dong^1*^**

^1^Department of Pathophysiology, College of Basic Medical Sciences, Jilin University, Changchun 130021, China.

**^*^**Corresponding author (email: [dongdl@jlu.edu.cn](mailto:dongdl@jlu.edu.cn))

**1 The similarities and differences between cytoplasmic translation and mitochondrial translation**

**Similarities**: (1) The process of mitochondrial translation is similar to that of cytoplasmic translation and proceeds through four steps: translation initiation, translation elongation, translation termination, and ribosome recycling [1,2]. (2) Translation in both the mitochondria and cytoplasm requires the involvement of tRNA, ribosomes, and other protein factors. Similar to cytoplasmic translation, cytoplasmic 80S ribosomes and mitochondrial 55S ribosomes comprise two subunits. The cytoplasmic ribosome comprises a 40S small subunit and a 60S large subunit. The 40S subunit comprises 18S ribosomal RNA (rRNA) and 33 cytoplasmic ribosomal proteins (CRP), which decode mRNA via aminoacyl-tRNA (tRNA) interactions. The 60S subunit comprises 5S, 5.8S, and 28S rRNA and 47 CRP, which catalyze peptide bond formation through peptidyl transferase reaction [3]. The mitochondrial ribosome located in the mitochondrial matrix consists of a large 39S subunit, which participates in catalyzing the peptidyl transferase reaction, and a small 28S subunit, which provides a platform for mRNA binding and decoding. The 39S large subunit comprises 16S mitochondrial rRNA (mt-rRNA) and 50 mitochondrial ribosomal proteins (MRP), while the 28S subunit comprises 12S mt-rRNA and 29 MRPs [4]. (3) Both mechanisms are based on the triplet codon code. All proteins involved in mitochondrial translation, including those required for ribosome biogenesis and assembly, are nuclear-encoded. This mechanism underscores the importance of mitochondrial-encoded protein translation and the need for effective coordination between nuclear and mitochondrial gene expression to promote mitochondrial protein synthesis.

**Differences**: (1) The ratio of RNA to protein in the two translation systems is different, with a lower ratio of RNA to protein in the 55S mitochondrial ribosome [5]. (2) Cytoplasmic and mitochondrial translation use different codons. The decoding mechanism for mitochondrial protein translation is simplified, with only 22 tRNAs. Mitochondria use the codons AGG and AGA for arginine, UAA and UAG as stop codons, and UGA as the codon for tryptophan [6]. (3) Due to the significant differences between mRNA in the cytoplasm and mitochondrial mRNA, there are differences in protein translation processes in the cytoplasm and mitochondria. (4) Cytoplasmic proteins are transcribed in the cell nucleus and translated in the cytoplasm, while mitochondrial transcription and translation occur in the same compartment, the mitochondrial matrix.

**2 Translation of cytoplasmic proteins**

Translation initiation is a rate-limiting step in protein synthesis. During the initiation phase of cytoplasmic translation, the eIF4F complex (including cap-binding protein eIF4E, scaffolding protein eIF4G, and RNA helicase eIF4A) binds to the 7-methylguanosine cap at the mRNA 5′ end. eIF4G also binds to the poly(A)-binding protein PABP at the 3′ end to promote circularization, which enhances translation initiation. The ternary complex of GTP-bound eIF2 and methionyl-tRNAi (tRNAiMet) binds to the 40S ribosomal subunit to form the 43S pre-initiation complex, which is recruited to the mRNA cap in an eIF4F-dependent manner to form the 48S initiation complex. The 5′ untranslated region (UTR) is scanned until the start codon AUG is recognized. Upon recognition, GTP hydrolysis occurs in the ternary complex, and the 60S ribosomal subunit joins the 48S initiation complex. GTP-bound eIF5B facilitates the joining of the 60S subunit, forming the functional 80S complex ready for the elongation phase. In cytoplasmic translation elongation, amino acids are added to a growing polypeptide chain in a multi-step process that involves codon-anticodon base pairing between mRNA codons and their corresponding aminoacyl-tRNAs. This process requires eukaryotic elongation factor 1A (eEF1A) for decoding codons and eEF2 to assist in the movement of the ribosome along the mRNA. In the termination phase of cytoplasmic translation, translation stops when a stop codon (UAA, UAG, or UGA) enters the ribosomal A site, releasing the deacylated tRNA. The ribosomal subunits dissociate into 40S and 60S subunits, which are then recycled for translation of other mRNAs.

**3 Mitochondrial Protein Translation**

Unlike cytoplasmic translation, mitochondrial mRNA does not contain a 5′UTR or has a very short 5′UTR and lacks cis-acting elements and introns [7]. Mitochondrial translation is dependent on various nuclear-encoded regulatory proteins. mtIF2 and mtIF3 regulate translation initiation, where mtIF3 positions the AUG and AUA start codons of mitochondrial mRNA at the peptidyl (P) site of the mitochondrial ribosome small subunit (mt-SSU) to initiate translation, and prevents premature association of mtLSU with mtSSU [8], while mtIF2 guides the binding of fMet-thionine tRNA to mRNA, serving as the signal for translation initiation and directing the assembly of mitochondrial large subunit and translation initiation [9]. Protein factors in the mitochondria bind to mitochondrial mRNA to affect gene expression; for example, mitochondrial mRNA recruits RNA-binding proteins TACO1, MITRAC, or C12orf62 to regulate the translation of complex IV [10]. Mitochondrial elongation, however, occurs at the peptidyl transferase center of mt-LSU, where mitochondrial elongation factor Tu (EFTu) forms a complex with GTP and aminoacyl-tRNA, guiding tRNA to the A site, and then the tRNA base pairs with the codon-anticodon site of mRNA, followed by mitochondrial elongation factor G1 (EFG1) binding to the ribosome at the A site, inducing the movement of A and P-tRNA to P and E sites, promoting ribosome translocation along the mRNA to translate subsequent codons [11,12]. Mitochondrial translation termination is recognized by mitochondrial translation release factor 1 (MTRF1) when a stop codon (UAA/UAG) is encountered, which promotes hydrolysis of the ester bond between peptidyl-tRNA and the C-terminal amino acid of the nascent polypeptide chain [13,14]. Once mitochondrial translation terminates, mitochondrial ribosome recycling factor (MRRF) and mitochondrial elongation factor (mtEFG2) promote the recycling of mitochondrial ribosomes, releasing uncharged tRNA by binding to the ribosome at the peptidyl site with deacylated tRNA. The specificity of mitochondrial-encoded protein translation reflects the possible timely and appropriate regulation of mitochondrial protein translation from the perspective of mitochondrial translation regulation to rapidly adapt to environmental energy demands. The induction of mitochondrial biogenesis associated with exercise in mouse skeletal muscle is accompanied by the expression of mitochondrial translation factors, further highlighting the important role of mitochondrial protein translation in mitochondrial biogenesis and function [15].

**Reference**

1. D'Souza AR, Minczuk M. Mitochondrial transcription and translation: overview. Essays Biochem. 2018; 62(3): 309-320.
2. Mai N, Chrzanowska-Lightowlers ZM, Lightowlers RN. The process of mammalian mitochondrial protein synthesis. Cell Tissue Res. 2017; 367(1): 5-20.
3. Thomson E, Ferreira-Cerca S, Hurt E. Eukaryotic ribosome biogenesis at a glance. J Cell Sci. 2013; 126(Pt 21): 4815-4821.
4. O'Brien TW. Properties of human mitochondrial ribosomes. IUBMB Life. 2003; 55(9): 505-513.
5. Greber BJ, Bieri P, Leibundgut M, Leitner A, Aebersold R, Boehringer D, et al. Ribosome. The complete structure of the 55S mammalian mitochondrial ribosome. Science. 2015; 348(6232): 303–8.
6. Anderson S, Bankier AT, Barrell BG, de Bruijn MH, Coulson AR, Drouin J, et al. Sequence and organization of the human mitochondrial genome. Nature. 1981; 290(5806):457–65.
7. Temperley RJ, Wydro M, Lightowlers RN, Chrzanowska-Lightowlers ZM. Human mitochondrial mRNAs--like members of all families, similar but different. Biochim Biophys Acta. 2010; 1797(6-7): 1081-5.
8. Münch C, Harper JW. Mitochondrial unfolded protein response controls matrix pre-RNA processing and translation. Nature. 2016; 534(7609): 710–3.
9. Spencer AC, Spremulli LL. The interaction of mitochondrial translational initiation factor 2 with the small ribosomal subunit. Biochim Biophys Acta. 2005; 1750(1): 69–81.
10. Szklarczyk R, Wanschers BF, Cuypers TD, Esseling JJ, Riemersma M, van den Brand MA, et al. Iterative orthology prediction uncovers new mitochondrial proteins and identifies C12orf62 as the human ortholog of COX14, a protein involved in the assembly of cytochrome c oxidase. Genome Biol. 2012; 13(2): R12.
11. Christian BE, Spremulli LL. Mechanism of protein biosynthesis in mammalian mitochondria. Biochim Biophys Acta. 2012; 1819(9–10): 1035–54.
12. Ott M, Amunts A, Brown A. Organization and Regulation of Mitochondrial Protein Synthesis. Annu Rev Biochem. 2016; 85: 77–101.
13. Lind C, Sund J, Aqvist J. Codon-reading specificities of mitochondrial release factors and translation termination at non-standard stop codons. Nat Commun. 2013; 4: 2940.
14. Soleimanpour-Lichaei HR, Kühl I, Gaisne M, Passos JF, Wydro M, Rorbach J, et al. mtRF1a is a human mitochondrial translation release factor decoding the major termination codons UAA and UAG. Mol Cell. 2007; 27(5): 745–57.
15. Yokokawa T, Kido K, Suga T, Isaka T, Hayashi T, Fujita S. Exercise-induced mitochondrial biogenesis coincides with the expression of mitochondrial translation factors in murine skeletal muscle. Physiol Rep. 2018; 6(20): e13893.
